# Changes in the Immunity, Histopathology, and Metabolism of Crayfish (*Procambarus clarkii*) in Response to Drought

**DOI:** 10.3390/ani12070890

**Published:** 2022-03-31

**Authors:** Hui Xu, Xuexia Bai, Yu Li, Jiajia Li, Yong Meng, Zhiqiang Xu, Jianqing Tang, Yan Lu, Yahong Huang

**Affiliations:** 1 School of Life Sciences, Nanjing University, Nanjing 210046, China; 505576242yi@sina.com (H.X.); xuexiabai@foxmail.com (X.B.); xxlyu@foxmail.com (Y.L.); luyan@nju.edu.cn (Y.L.); 2 Freshwater Fisheries Research Institute of Jiangsu Province, Nanjing 210017, China; jslijj@163.com (J.L.); mengy6@163.com (Y.M.); zhiqiangx@163.com (Z.X.); jstjq@163.com (J.T.)

**Keywords:** drought, histopathology, immunity, metabolomics, *Procambarus clarkii*, ROS

## Abstract

**Simple Summary:**

The freshwater biodiversity crisis is in the spotlight due to the destruction of freshwater ecosystems. Factors threatening freshwater biodiversity are much wider and more complex, including climate change and severe weather events such as drought, unexpected floods, heavy storms, etc. Drought is an important factor contributing to this crisis. In this study, we evaluated changes in the immune function, antioxidant function, histopathology, and metabolites of crayfish in response to drought. The results indicate that drought suppresses immune function, the balance between oxidative and antioxidative systems, and induces tissue damage and metabolic disorder. Our work provides more information on how crayfish respond to drought.

**Abstract:**

Freshwater ecosystems are among the most threatened ecosystems on Earth. The freshwater biodiversity crisis has caused widespread global concern. Drought as one of the factors causing freshwater biodiversity is still poorly understood. Crayfish is often used in academic research as a biological indicator. In this study, flow cytometry, hematoxylin-eosin staining, and untargeted metabolomics were used to analyze the immune function, histopathology, and metabolism of crayfish under drought conditions. After drought exposure, the total hemocytes count (THC) was significantly decreased (from 8.9 × 10^5^ mL^−1^ in the control group to 2.2 × 10^5^ mL^−1^ at day 5). Phagocytosis decreased by 66% after 5 days of drought. The level of reactive oxygen species (ROS) in the hepatopancreas was upregulated. Moreover, histological disorder and metabolism changes in the hepatopancreas were obvious. These results indicate that drought suppresses immune function, disrupts the balance of oxidative and antioxidative systems, and induces tissue damage and metabolic changes in crayfish.

## 1. Introduction

The proportion of freshwater in the world’s water supply is extremely small, at just 0.01%. However, this tiny source of fresh water supports the survival of a vast array of species across the globe [1]. With the increasing demand for freshwater resources, freshwater ecosystems are among the most threatened ecosystems on earth. The freshwater biodiversity crisis includes species loss and the breakdown of ecological processes and resources [2], and has become the focus of research attention [3]. A wide array of factors threaten freshwater biodiversity, including invasive alien species (IAS), climate change, and severe environmental conditions. Long-standing drought is among the severe weather events that threaten freshwater biodiversity [4]. Indeed, the impact of drought in freshwater biodiversity remains poorly understood. There are reports of the response, adaptation, conservation status, and distribution of freshwater taxa under drought conditions [5,6,7,8]. However, the effects on the immunity, antioxidation, histopathology, and metabolism of freshwater taxa remain unclear.

Crayfish are keystone organisms in aquatic ecosystems [9]. The red swamp crayfish *Procambarus clarkii* is often designated as a bioindicator of aquatic conservation and environmental stress due to its wide, cosmopolitan distribution, long life-span, suitability for laboratory experiments, and high sensitivity to natural and chemical stimuli [10]. Crayfish are among the most important groups of invasive species. They alter the structure and function of nonnative ecosystems and contribute to the freshwater biodiversity crisis [11].

The immune system is essential for crayfish survival and coping with environmental stress and information about the crayfish immune system is limited [12]. Crayfish immunity has received great attention in recent years [13]. Hemocytes are key participants in the immune responses and the total hemocytes count (THC) is often used as an important indicator to reflect the immunological status of crayfish [14]. THC is very susceptible to environmental stress, so it can serve as an important indicator of whether crayfish are under adverse stress. Under normal conditions, a dynamic balance in the production and removal of reactive oxygen species (ROS) is maintained, a balance that is disrupted under adverse conditions [15,16]. An unfavorable consequence of environmental stress is the overproduction and subsequent oxidative damage to organisms. Excessive ROS levels cause protein dysfunction, lipid peroxidation, and DNA damage, ultimately leading to oxidative damage with potential tissue injury or cell death [17,18,19]. Thus, an effective antioxidant response to maintain the balance between oxidation and antioxidation is critical for the function and survival of cells and tissues. The antioxidant defense system is composed of antioxidant enzymes and non-enzymatic cellular antioxidants, such as superoxide dismutase, catalase, vitamin E, and scavengers of hydroxyl radicals [20].

Metabolism is a series of chemical reactions that sustain the life of a living organism [21]. Metabolites, the products of metabolism, have a wide range of functions in cells, such as in providing energy and enabling signal transduction. Glucose and fatty acids provide the majority of cellular energy in animals. It has also been reported that metabolites participate in cell signal transduction by interacting with proteins [22,23,24]. Metabolites such as ATP, acetyl-CoA, and NAD+ can change protein activity by regulating post-translational modifications [25,26,27]. Furthermore, metabolites, as the end products of cell functions, are highly sensitive to environmental changes [28]. Thus, it is important to identify and characterize metabolites and metabolic pathways [29]. Metabolomics is a fundamental and powerful technique for the comprehensive study of metabolites. In this work, we applied untargeted metabolomics to reveal the effect of drought on crayfish metabolism. Untargeted metabolomics analysis focuses on obtaining data for as many species as possible, annotating metabolites, and reviewing both known and unknown metabolic changes [29]. When performed together with high-resolution mass spectrometer, untargeted metabolomic analysis is effective in examining the relationships between interrelated metabolites from multiple pathways [30].

In this study, we aimed to assess changes in the immunity, antioxidation, histopathology, and metabolite changes of crayfish and to gain deeper insight into the mechanisms of their response to drought exposure for 1, 3, and 5 days.

## 2. Materials and Methods

### 2.1. Experimental Animals and Drought Exposure

The crayfish were obtained from a local crayfish farm and were kept in plastic boxes at room temperature and fed once daily with commercial pellets. Crayfish were placed under the conditions of 24 h light–dark cycles (LD 12:12) and temperatures of 20 ± 2 °C. The crayfish were allowed an adjustment period of 7 days, before individual crayfish of similar size (9.83 ± 0.84 cm in length, 31.97 ± 9.10 g in wet weight) were then selected for the drought exposure experiments. Crayfish were kept in boxes with a mixture of sand and garden soil to simulate drought. The drought exposure time was 1, 3, and 5 days. In the metabolomics and survival analysis, there were 10 individuals each in the control and the drought groups. For all other experiments, there were 5 individuals in each group.

### 2.2. Total Hemocytes Count

Hemolymph samples were collected from the control group and the drought groups with anticoagulant solution (27 mM sodium citrate, 336 mM NaCl, 115 mM glucose, 9 mM EDTA, pH 7). Hemolymph was centrifuged at 1000× *g* for 5 min at 4 °C to collect the hemocytes. Hemocytes were then washed twice and resuspended in ice-cold crayfish-saline (CFS; 0.22 M NaCl, 5.4 mM KCl, 2.6 mM MgCl_2_, 2 mM NaHCO_3_, pH 7.4) [31]. Total hemocyte counts were analyzed on NovoCyte flow cytometer systems.

### 2.3. Phagocytosis Assay

Hemolymph was collected from crayfish in the drought and control groups using the method described in Section 2.2. Each group contained 5 individuals. A phagocytosis assay was implemented using fluorescent red latex beads (1 μm diameter, L-2778, Sigma-Aldrich, St. Louis, MO, USA). Before beginning this assay, latex beads were added to CFS and placed in an incubator at 37 °C for pre-warming; the pre-warmed beads were then incubated with hemocytes for 4 h at 37 °C. Pre-cooled CFS was added to terminate phagocytosis. Hemocytes were harvested and analyzed using NovoCyte flow cytometer systems (PE channel).

### 2.4. ROS Detection

Single-cell hepatopancreas tissue suspension was prepared before ROS detection. The hepatopancreases of crayfish were diced, mashed, and resuspended with CFS. Then, the product was passed through a 40-μm cell strainer (CSS010040, BIOFIL, Guangzhou, China). Cells were collected after 5 min centrifugation at 1000× *g*. The cells were then washed and resuspended with CFS. ROS detection was performed using the DCFH-DA oxidation method according to the protocol of the detection kit (S0033S, Beyotime, Shanghai, China). DCFH-DA was added to the cell suspension at a ratio of 1:1000. Fluorescent probes were allowed to diffuse into cells during a 20-min incubation at 37 °C. Afterwards, extra DCFH-DA was removed by washing with ice-cold CFS. ROS levels were determined on the NovoCyte flow cytometer systems (FITC channel).

### 2.5. Hematoxylin and Eosin Staining

Hepatopancreas tissues were immediately fixed with 4% paraformaldehyde solution after being removed from the crayfish. The samples were cut into 4-μm-thick sections using a microtome (RM2016, Laike, Xiangfan, China) and stained by hematoxylin and eosin (H&E) according to routine protocols including dewaxing, hematoxylin staining, eosin dye staining, and sealing. Dewaxing was performed as follows: xylene I for 20 min, xylene II for 20 min, 100% ethanol I for 5 min, 100% ethanol II for 5 min, 75% ethanol for 5 min, and rinsing with tap water. After dewaxing, sections were stained with hematoxylin solution for 3–5 min, treated with hematoxylin differentiation solution and with Scott’s Tap Water Substitute for hematoxylin bluing, and then rinsed with tap water. The sections were then stained with eosin dye for 5 min. The last step was to dehydrate and seal with neutral gum. The tissue sections were examined and photographed with an upright optical microscope (Nikon Eclipse E100, Nikon, Tokyo, Japan).

### 2.6. Metabolomics Analysis

#### 2.6.1. Metabolite Extraction

Hepatopancreases were collected from crayfish for metabolomics analysis and were immediately stored in a freezer at −80 °C. There were 10 replicated samples in each group (the 5-day drought and control groups). Frozen samples were placed on the dry ice and cut into pieces, then 400 μL of extract solution (methanol/acetonitrile, =3:1, (*v*/*v*)) and 2 steel balls were added to the samples to grind the samples. After grinding with a grinder, the samples were set down for 1 h at 4 °C, centrifuged at 12,000 rpm for 15 min at 4 °C, and then concentrated in vacuum to dryness. Then, 100 μL of 50% methanol solution (methanol/water, =1:1, (*v*/*v*)) was added to the samples for reconstitution. The samples were vortexed at 2000 rpm for 3 min at 4 °C and centrifuged at 12,000 rpm for 15 min at 4 °C. The resulting supernatant was used for injection analysis. An equal volume of supernatant was taken from all samples to prepare a quality control (QC) sample.

#### 2.6.2. Liquid Chromatograph Conditions

An Ultra-High-Performance Liquid Chromatograph (UHPLC) and chromatographic column (ACQUITY UPLC HSS T3 1.7 µm, 2.1 mm × 150 mm column; Waters, Framingham, MA, USA) were employed for chromatographic analysis. The working pattern of the chromatographic column was set as follows: temperature maintained at 40 °C, flow rate 0.25 mL/min, and injection sample volume 5 μL. The mobile phase consisted of 5 mM ammonium formate (A), acetonitrile (B), 0.1% formic acid (C), and acetonitrile + 0.1% formic acid (D). The chromatographic gradient elution procedure is shown in Table 1. A quality control sample was set up every 5 test samples to monitor and analyze the system stability and the reliability of experimental data.

#### 2.6.3. Mass Spectrometry Conditions

Mass spectrometry was performed on a Thermo QE HF-X mass spectrometer. Electrospray ionization (ESI) mass spectrometry (MS) was employed for detection in both positive and negative ion modes. The ESI source conditions were as follows: ion source—ESI ion source; sheath gas flow rate—30 arb; aux gas flow rate—10 arb; spray voltage—2.5 kV (+)/2.5 kV (−); S-Lens RF—50%; capillary temp—352 °C; aux gas temp—300 °C; collision energy (NCE)—30, top *n* = 8; scanned *m*/*z* range—70–1050; analysis software—Xcalibur version 4.1.

#### 2.6.4. Statistical Analysis

Peak alignment, retention time correction, and peak area extraction were performed on the original data using the Compound Discovery software. Metabolite identification was performed by means of exact mass match (<10 ppm), secondary spectra match, and database search. R statistical software was used for multidimensional statistical analysis including partial least-squares discrimination analysis (PLS-DA) and orthogonal PLS-DA (OPLS-DA). These analyses and information retrieval were conducted on Compound Discoverer™ 3.1 (Thermo Scientific™, Waltham, MA, USA). Pathway analysis was performed with the MATLAB scripting language (Mathworks^®^, Natick, MA, USA), using the MetaboAnalyst (http://www.metaboanalyst.ca/) and KEGG (http://www.genome.jp/kegg/) websites, both accessed on 4 March 2022.

### 2.7. Statistical Analysis

Data are presented as mean ± standard error of the mean (SEM). Statistical analysis was performed by Student’s *t*-test when only two value sets were compared. One-way ANOVA was used when the data involved three or more groups. The different letters indicate significant differences between drought times (*p* < 0.05).

## 3. Results

### 3.1. Drought Suppresses Immune Function

**Figure 1:**
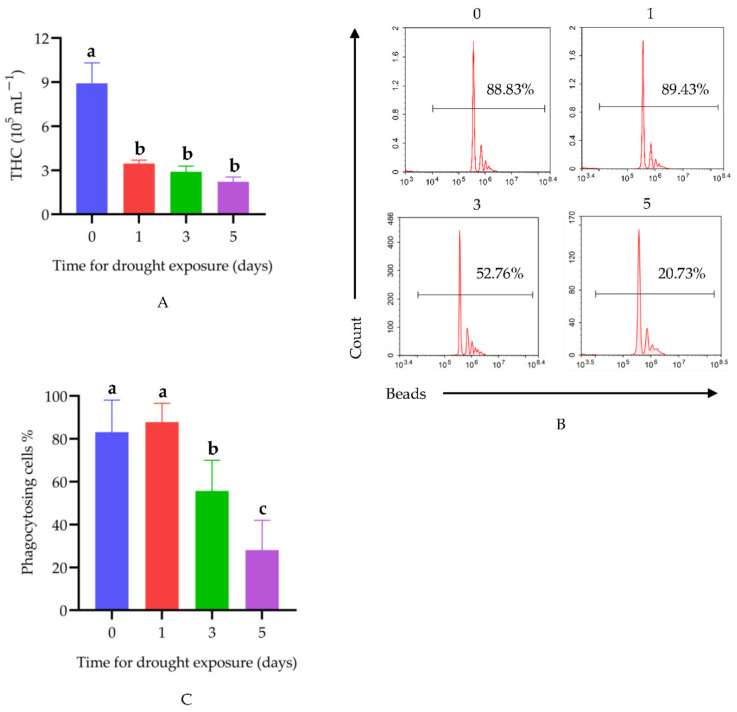
Effects of drought on immune functions in hemocytes: (**A**) THC; (**B**) flow cytometry analysis of phagocytic ability; (**C**) statistical results of phagocytosis assay. Data are represented as mean ± SEM (*n* = 5). Different lowercase letters on the bar chart indicate the significant differences between different drought times (*p* < 0.05).

Figure 1A shows the THC changes in crayfish under drought exposure. The THC in the control group was 8.92 × 10^5^ mL^−1^. After 1, 3, and 5 days of drought, the THC was 3.46 × 10^5^, 2.90 × 10^5^, and 2.21 × 10^5^ mL^−1^, respectively. Compared with the control group crayfish, the THC in crayfish exposed to drought decreased rapidly. In the drought groups, there was no significant difference at 1, 3, and 5 days under drought exposure. Phagocytosis was also detected to evaluate the immune function. After 3 and 5 days drought exposure, the phagocytic ratio of hemocytes decreased by 33% and 66%, respectively. However, after 1 day of drought, there was no significant difference in the phagocytosis rate (Figure 1B,C).

### 3.2. Drought Promotes Overproduction of ROS in Hepatopancreas

**Figure 2:**
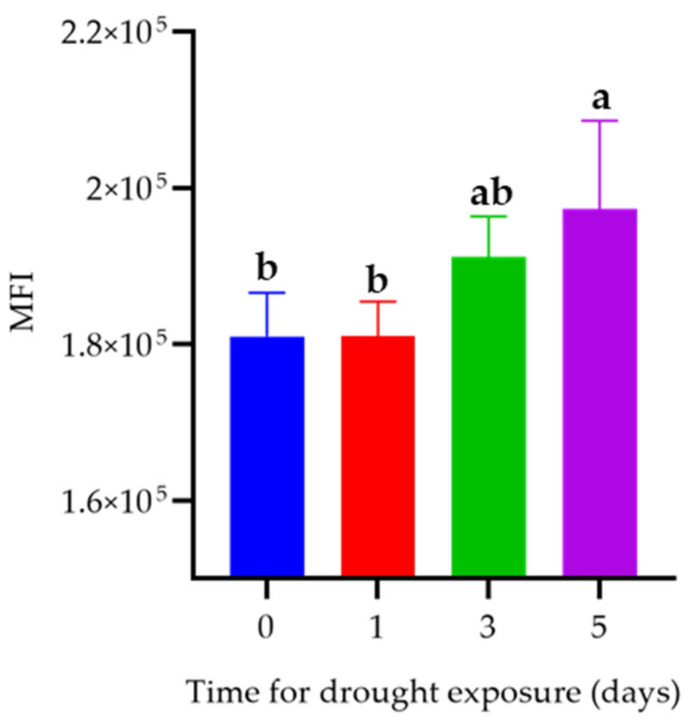
Effect of drought on ROS level in hepatopancreas. Mean fluorescence intensity (MFI). Data are represented as mean ± SEM (*n* = 5). Different lowercase letters on the bar chart indicate the significant differences between different drought times (*p* < 0.05).

Compared with hepatopancreas from control group crayfish, the ROS level in hepatopancreas dramatically increased in the group exposed to 5 days of drought. However, the ROS level was not statistically increased after drought exposure for 1 and 3 days (Figure 2). 

### 3.3. Drought Induces Histological Disorder in Hepatopancreas

**Figure 3:**
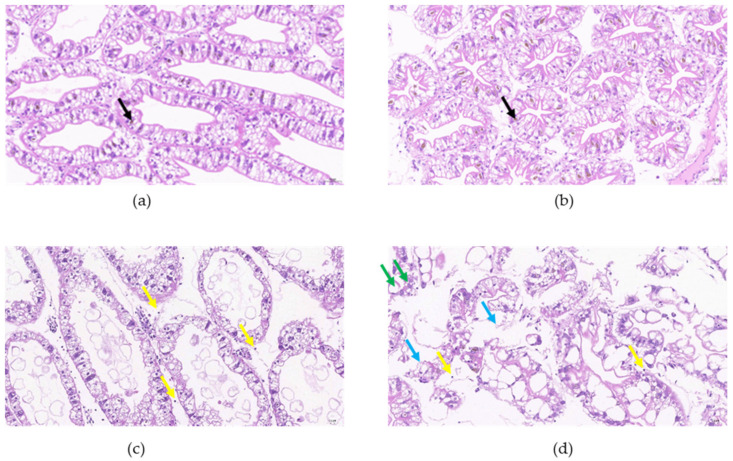
Histological sections of hepatopancreas after drought. (**a**) Control and drought exposure for (**b**) 1, (**c**) 3, and (**d**) 5 days. Vacuole with yellow pigment (black arrow), inflammatory cell (yellow arrow), oozing protein fluid (blue arrow), vacuole (marked by green arrow). H&E stain (200×).

The hepatopancreases of control crayfish were obviously well organized in structure. The hepatic tubules were composed of a variety of different hepatocytes. R cells accounted for the largest proportion of hepatocytes, and fat was abundant in the cytoplasm. Vacuoles with yellow pigment could be seen in the cytoplasm of F cells. The tubule lumens were well organized and had an asterisk-like appearance. Exudation and inflammatory cell infiltration were not observed in the intercellular substance (Figure 3a). After drought exposure for 1 day, the hepatopancreas structures remained well organized (Figure 3b).

The hepatopancreas structures of crayfish exposed to drought for 3 days were different from those of control samples, with evidence of hepatic tubule expansion and tubule lumen dilatation. Additionally, epithelial cells became shorter, and more tissue fragments appeared in lumens. Yellow pigment could be seen in the cytoplasm of individual F cells. Specifically, a small amount of oozing protein fluid was seen in the local intercellular substance and inflammatory cell infiltration was present (Figure 3c).

After 5 days of drought exposure, the hepatopancreases of crayfish were observed to have abnormal structures. The foamy cytoplasm of R cells disappeared, and more round vacuoles of different sizes were seen. Inflammatory cell infiltration was obvious in the hepatopancreases, the hepatic tubule structures were markedly disorganized, and some tubules were damaged and ruptured (Figure 3d).

### 3.4. Drought Induces Metabolic Changes in Hepatopancreas

#### 3.4.1. Quality Control (QC) Analysis

Total ion chromatography (TIC) spectral comparison of QC samples was used to appraise the stability of the system in positive and negative ion modes. The overlapping total ion current chromatograms showed that the response intensity and retention time of all spectral peaks basically overlapped, indicating that the variation caused by instrumental errors during the whole experiment was relatively small, and thus, that the data were reliable. The response peak height difference of the internal standard was used to determine whether the detection was stable. The retention time and response intensity of the internal standard were stable, indicating that the stability of the data collection was excellent. Blank samples were detected to analyze substance residue in the detection process. No obvious peak of the internal standard was detected in the blank samples, indicating that the substance residue was well controlled and cross-contamination between samples was within a controllable range.

#### 3.4.2. Metabolite Profile of Hepatopancreas

**Figure 4:**
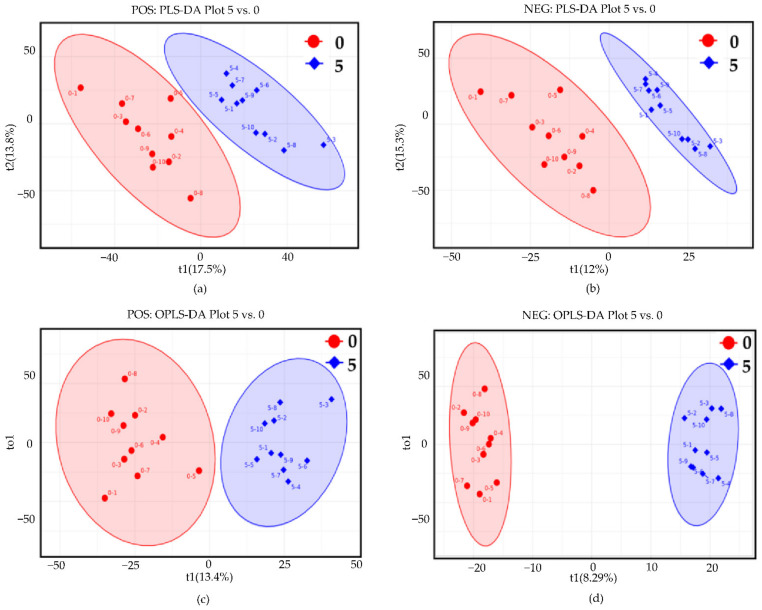
Partial least squares-discriminant analysis (PLS-DA) and orthogonal PLS-DA (OPLS-DA) score plots of hepatopancreas samples. (**a**,**b**) PLS-DA score plots in positive and negative ion mode, respectively; (**c**,**d**) OPLS-DA score plots in positive and negative ion mode, respectively. Red represents control group; blue represents drought group (5 days). Each point represents a sample.

**Figure 5:**
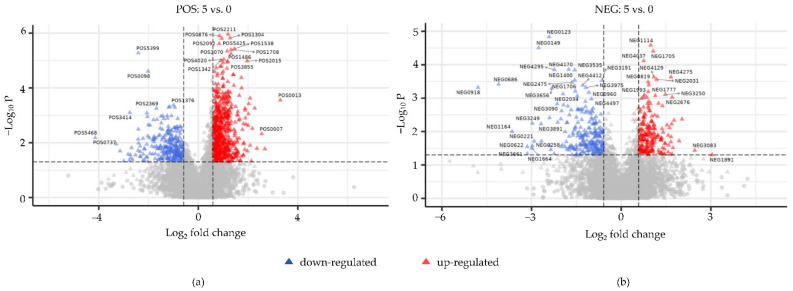
Volcano plots of hepatopancreas samples in (**a**) positive and (**b**) negative ion modes. Red and blue triangles represent significantly upregulated and downregulated metabolites, respectively (|FC| ≥ 1.5 and *p* value < 0.05).

Detection of small molecule metabolites in hepatopancreas was performed using liquid chromatography-tandem mass spectrometry (LC-MS/MS) in positive and negative ion modes. A total of 5493 and 4579 metabolites were identified in hepatopancreas in the positive and negative ion modes, respectively. The detected metabolites were then subjected to metabolic difference analysis. The results of partial least squares-discriminant analysis (PLS-DA) and orthogonal PLS-DA analysis (OPLS-DA) showed strong separation between the drought group and the control group, demonstrating that the hepatopancreas samples had great repeatability and consistency in the same group, and there were evident differences between the two groups (Figure 4). Univariate analysis was used to determine the significance of metabolite changes between the two sets of samples, so as to screen for potential marker metabolites (|FC| ≥ 1.5 and *p* value < 0.05 as the screening criteria), and the variance importance in projection (VIP) value was used to perform auxiliary screening with VIP > 1 as the screening criterion. Volcano plots suggested that 641 metabolites were significantly upregulated and 283 metabolites were significantly downregulated in positive ion mode (Figure 5a). In addition, 200 metabolites were significantly upregulated and 237 metabolites were significantly downregulated in negative ion mode (Figure 5b). Detailed information on the significantly altered metabolites is given in Table S1.

#### 3.4.3. Identification of Potential Biomarkers and Analysis of Related Metabolic Pathways

**Figure 6:**
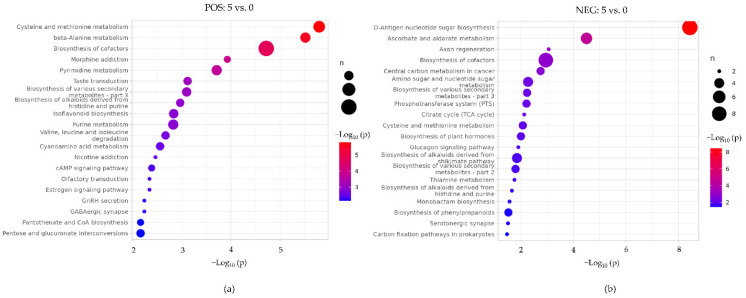
KEGG metabolic pathway enrichment bubble map between drought and control groups: (**a**) positive ion mode; (**b**) negative ion mode. Bubble sizes represent the numbers of metabolites in metabolic pathways whose levels were found to be altered. Bubble colors represent *p*-values from low (blue) to high (red).

In order to show the differences in the expression patterns of metabolites, accurately screen marker metabolites, and conduct research on changes in related metabolic pathways, we performed hierarchical clustering on each group of samples. When the selected candidate metabolites were reasonable and accurate, samples from the same group could be present in the same cluster. Meanwhile, metabolites in the same cluster had similar expression patterns and may have been relatively close in the reaction steps of the metabolic process. Hierarchical clustering of hepatopancreas metabolites in the drought and control groups showed an obvious difference in the metabolite prolife. Meanwhile, metabolism patterns among samples in the same group were similar (Figure S1). Twenty-four metabolites were characterized as potential crayfish biomarkers of the response to drought. The corresponding information and the related metabolic pathways of these biomarker candidates are shown in Table 2. The biomarker quantitative analysis results are shown in Figure S2. Kyoto Encyclopedia of Genes and Genomes (KEGG) enrichment analysis showed that the differential metabolites were mainly enriched in the pathways of amino acids (cysteine, methionine, and beta-alanine) metabolism, and the biosynthesis of various secondary metabolites and cofactors (Figure 6). These changes are represented by the color and size on the bubble. Bubble colors represent *p*-values from low (blue) to high (red) and bubble sizes represent the numbers of metabolites in metabolic pathways whose levels were found to be altered. After drought exposure, the abundance of metabolites involved in cysteine and methionine metabolism, such as L-glutathione (L-GSH), S-adenosylmethionine (SAM), and S-glutathione-L-cysteine, was reduced. These metabolites are sensitive to ROS and, therefore, possess antioxidant capacity. The antibacterial secondary metabolites roseoflavin and dapdiamides were downregulated. The cofactor biotin was also reduced, which has important implications for immunity.

## 4. Discussion

In this study, we confirmed that drought can weaken the immune function of crayfish. As an important indicator of hemocytes function, THC is often used to assess the immune function of crayfish [32,33]. Our results indicated that THC was significantly decreased in the control group (8.9 × 10^5^ mL^−1^) compared to the 1-day drought group (3.5 × 10^5^ mL^−1^). Although there was no significant difference in THC among the drought groups, it showed a downward trend with the increasing of the drought period. The THC results indicated that a short period of drought could lead to a significant decrease in THC, and as the drought period continued, the decline in THC slowed down. Crayfish lack an adaptive immune system and rely mainly on innate immunity [34]. Phagocytosis is one of the fundamental cellular immune defense parameters, and helps to eliminate invading pathogens. Phagocytosis of foreign pathogens is mainly completed by hemocytes [33,35]. Phagocytosis was found to be reduced after drought exposure for 3 and 5 days, but not for 1 day. Particularly, phagocytosis decreased by 66% after 5 days of drought. According to these results, we infer that drought suppresses the immune function of crayfish, and the decline in phagocytic ability may be a possible reason.

Excessive accumulation of ROS caused by environmental stress could result in oxidative stress, leading to lipid peroxidation, DNA damage, and tissue injury. Thus, an effective antioxidant response is essential for aquatic organisms to cope with oxidative stress [36,37,38]. In our study, we found increased ROS levels in hepatopancreas after 5 days of drought, but not after exposure for only 1 or 3 days. ROS also serves as a defense mechanism against microbial infection and plays a role in multiple immune responses [39]. The innate immune system is the first line of host defense, and one important mechanism is the production of ROS [40]. Phagocytic cells release large amounts of ROS to cope with microbial infections, a process referred to as respiratory burst or oxidative burst. Therefore, the increase in ROS after drought exposure is more likely due to the decrease in antioxidant capacity. The responses of the immune system and antioxidant defense may potentially be related. The mechanism of ROS production and the effects of ROS on crayfish under drought conductions need further research.

Histological disorder is a higher-level biological organization response, reflecting prior alteration in physiological and/or biochemical functions [41]. Environmental stress can cause tissue damage, which has been widely reported [42,43]. A histopathological examination is a fundamental and useful tool to study the pathological processes of organisms under environmental stress [41,44]. In our study, structural damage to the hepatopancreas was severe with prolonged drought. Hepatic tubule rupture and tubule lumen dilatation were clearly observed. Moreover, tissue fragments in lumens are compelling evidence of impaired hepatic tubule integrity. Tissue damage and inflammation are orchestrated by the infiltration and activation of various immune cells. After drought exposure for 3 and 5 days, the appearance of immune cell infiltration indicated acute inflammation in the hepatopancreas. These results, combined with the ROS results, suggest that drought may potentially cause tissue damage to the hepatopancreas, and ultimately affect the physiological functions of crayfish.

The results of the hepatopancreas metabolomics analysis showed that amino acids, secondary metabolites, and metabolite cofactors were alternated after drought exposure. Methionine and cysteine, as primary sulfur amino acids (SAAs), play crucial roles in protein structure, metabolism, oxidation, and immunity [45,46]. Cysteine is extremely antioxidative because of the high sensitivity to ROS [47]. The metabolites of cysteine and methionine, such as SAM, polyamines, taurine, and GSH, are essential for critical functions in crayfish. Methionine residues can bind to ROS and then convert to methionine sulfoxide (MetO), which inactivates ROS. Under the catalysis of methionine adenosyltransferase (MAT), methionine produces SAM, which increases cystathionine γ-synthase (CBS) activity, promotes cysteine synthesis, and ultimately upregulates the GSH level. Cysteine residues are also easily oxidized and the antioxidant capability of cysteine is primarily reflected in the products of GSH, hydrogen sulfide (H_2_S), and taurine. GSH is a prime cellular antioxidant. ROS readily oxidizes GSH to generate glutathione disulfide (GSSG) through the catalysis of GSH-Px. Then, GSSG is reduced to GSH by glutathione reductase. Consequently, the GSH/GSSG cycle is conducive to eliminating ROS and preventing oxidative damage. Cysteine is catabolized to generate hydrogen sulfide (H_2_S) through a series of desulfuration reactions [48]. It is well known that H_2_S is an extremely toxic substance for aerobic organisms, partially due to its ability to suppress mitochondrial electron transport chains by reacting with cytochrome c oxidase [49]. However, recent studies have revealed that H_2_S is an antioxidant agent that works to balance the oxidative and antioxidative systems. Taurine also demonstrates antioxidant capacity, by scavenging ROS, in many models. Our metabolomics results showed that the metabolites involved in cysteine and methionine metabolism, including L-GSH, SAM, and S-glutathionyl-L-cysteine, were downregulated after drought exposure, indicating that the antioxidant capacity of cysteine and methionine was decreased. This result was consistent with the elevated ROS level.

After exposure to drought, the biosynthesis of secondary metabolites was reduced, mainly those functioning as antibacterial compounds, including roseoflavin, dapdiamide A, dapdiamide B, and dapdiamide C. Roseoflavin, the only known natural riboflavin (vitamin B2) analogue with antibiotic activity, targets the FMN riboswitch (a riboflavin transporter) to block cell growth [50,51,52,53]. Dapdiamides are tripeptide antibiotics formed by unconventional amide ligases [54]. The reduction in antibacterial substances indicated that the crayfish were more susceptible to microbial infection under drought exposure. The content of cofactors was changed under drought. Biotin, a water-soluble B-complex vitamin, is well-known as being a cofactor for five indispensable carboxylases (pyruvate carboxylase, acetyl-CoA carboxylase 1, acetyl-CoA carboxylase 2, propionyl-CoA carboxylase, and methyl crotonyl-CoA carboxylase) [55]. Since biotin-dependent carboxylases are involved in a variety of cellular metabolic pathways, an abnormal biotin metabolism may be a key factor in immune and inflammatory diseases caused by biotin deficiency. Biotin deficiency affects the immunological functions of lymphocytes [56]. Biotin demands increase with cell proliferation and activation [57]. When an immune response is initiated, the rate of lymphocyte proliferation increases. Therefore, lymphocytes may be sensitive to the biotin status. In addition, biotin deficiency increases the expression of proinflammatory cytokine genes, such as interleukin 1β (IL-1β), tumor necrosis factor α (TNF-α), and interferon γ2 (IFN-γ2), and enhances the inflammatory response [58,59,60,61]. The decrease in biotin in crayfish after drought exposure may be further supporting evidence for reduced immune function and increased inflammatory hepatopancreas injury.

To disperse between isolated waterbodies, freshwater organisms usually use a variety of methods to cross terrestrial barriers. For an invasive species such as crayfish, in addition to flooding events, other animals, or anthropogenic activity, the ability to walk on land is advantageous as it facilitates spreading to other bodies of water. This is favorable behavior that is conducive to the invasion of crayfish [62]. Successful invasion by crayfish has been reported to be influenced by their immunity [63]. Although we found that their immunity reduced under drought conduction, there was no significant influence on their survival (Figure S3). The ability to survive under drought conditions may, therefore, be a contributing factor to successful crayfish invasion.

In addition to Japan and China, Europe and Africa also have territory occupied by crayfish. Thus, they have become an invasive biological species as well as a world-class food product. Our study found out that drought could suppress the immune function and disrupt the balance of oxidative and antioxidative systems, which might provide some clues for crayfish breeding and control technology.

## 5. Conclusions

Under drought conditions, the immunity, histopathology, and metabolism of crayfish were found to be altered. After drought exposure, THC and phagocytosis were decreased. The ROS levels were increased, and the hepatopancreas structure became disorganized. These results indicated a decline in immune function, an imbalance in the oxidant and antioxidant systems, and metabolic changes in crayfish after drought exposure. The metabolites that were altered in response were mainly amino acids, secondary metabolites, and cofactors. This study thus provides more information on the mechanisms by which crayfish cope with drought.

## Figures and Tables

**Table 1:** Gradient of mobile phase.

Time (min)	Flow Rate (mL/min)	Negative Ion Mode		Positive Ion Mode	
		A (%)	B (%)	C (%)	D (%)
0	0.25	98	2	98	2
1	0.25	98	2	98	2
9	0.25	50	50	50	50
12	0.25	2	98	2	98
13.5	0.25	2	98	2	98
14	0.25	98	2	98	2
17	0.25	98	2	98	2

**Table 2:** Identification of potential biomarkers and their related information.

No.	Name	Molecular Formula	Retention Time (min)	Mode (C18)	FC Value	*p* Value	VIP Value	Main Metabolic Pathways
1	4-Aminobutyric acid	C_4_H_9_NO_2_	1.23	Pos	−1.85	0.02	1.47	cAMP signaling pathway GABAergic synapse Estrogen signaling pathway GnRH secretion
2	Biotin	C_10_H_16_N_2_O_3_S	1.28	Pos	−1.52	0.05	1.50	Biotin metabolism Biosynthesis of cofactors
3	Dethiobiotin	C_10_H_18_N_2_O_3_	10.52	Pos	1.70	0.00	2.19	Biotin metabolism Biosynthesis of cofactors
4	D-O-Phosphoserine	C_3_H_8_NO_6_P	1.13	Pos	−2.09	0.01	1.79	Aminoacyl-tRNA biosynthesis Biosynthesis of various other secondary metabolites Biosynthesis of various antibiotics
5	Genistein	C_15_H_10_O_5_	13.95	Pos	9.82	0.00	1.94	Isoflavonoid biosynthesis Biosynthesis of secondary metabolites
6	Glycine anhydride	C_4_H_6_N_2_O_2_	1.79	Pos	−1.77	0.00	1.66	Pyrimidine metabolism beta-Alanine metabolism Pantothenate and CoA biosynthesis
7	Guanosine-5’-monophosphate	C_10_H_14_N_5_O_8_P	2.30	Pos	−2.60	0.01	1.44	Purine metabolism Biosynthesis of alkaloids derived from histidine and purine Olfactory transduction
8	L-Glutathione oxidized	C_20_H_32_N_6_O_12_S_2_	3.57	Pos	−2.44	0.01	1.51	Glutathione metabolism Biosynthesis of cofactors
9	Nicotinamide	C_6_H_6_N_2_O	2.21	Pos	−4.05	0.00	2.21	Nicotinate and nicotinamide metabolism Metabolic pathways Biosynthesis of cofactors
10	Ophthalmic acid	C_11_H_19_N_3_O_6_	4.56	Pos	−1.86	0.01	1.83	Cysteine and methionine metabolism Metabolic pathways
11	(R)-Prunasin	C_14_H_17_NO_6_	3.68	Pos	−5.32	0.00	2.26	Metabolic pathways Biosynthesis of secondary metabolites
12	S-Adenosylhomocysteine	C_14_H_20_N_6_O_5_S	3.78	Pos	−1.52	0.02	1.50	Cysteine and methionine metabolism Biosynthesis of cofactors”
13	S-Glutathionyl-L-cysteine	C_13_H_22_N_4_O_8_S_2_	1.25	Pos	−1.65	0.03	1.55	Cysteine and methionine metabolism Metabolic pathways
14	Tyramine	C_8_H_11_NO	4.79	Pos	−2.10	0.01	1.52	Isoquinoline alkaloid biosynthesis Biosynthesis of alkaloids derived from shikimate pathway Biosynthesis of secondary metabolites
15	Citric acid	C_6_H_8_O_7_	1.21	Neg	−2.90	0.00	2.33	Citrate cycle (TCA cycle) Biosynthesis of phenylpropanoids Biosynthesis of alkaloids derived from shikimate pathway
16	γ-Glutamyltyramine	C_13_H_18_N_2_O_4_	4.15	Neg	1.60	0.03	1.70	Metabolic pathways Biosynthesis of cofactors
17	5-Hydroxy-L-tryptophan	C_11_H_12_N_2_O_3_	4.23	Neg	−2.93	0.01	2.03	Biosynthesis of alkaloids derived from shikimate pathway Serotonergic synapse
18	Isonocardicin A	C_23_H_24_N_4_O_9_	6.24	Neg	−2.27	0.03	1.75	Monobactam biosynthesis Biosynthesis of secondary metabolites
19	Misonidazole	C_7_H_11_N_3_O_4_	1.20	Neg	−1.75	0.02	1.75	Biosynthesis of various antibiotics Biosynthesis of secondary metabolites
20	β-N-Acetylglucosamine	C_8_H_15_NO_6_	1.18	Neg	−1.52	0.03	1.75	Amino sugar and nucleotide sugar metabolism Phosphotransferase system (PTS)
21	Pantothenic acid	C_9_H_17_NO_5_	5.96	Neg	−1.66	0.01	2.03	Pantothenate and CoA biosynthesis Biosynthesis of secondary metabolites Biosynthesis of cofactors
22	S-(5-Deoxy-D-ribos-5-yl)-L-homocysteine	C_9_H_17_NO_6_S	1.21	Neg	−1.68	0.01	1.85	Cysteine and methionine metabolism Metabolic pathways
23	S-Glutathionyl-L-cysteine	C_13_H_22_N_4_O_8_S_2_	1.18	Neg	−1.81	0.01	2.02	Cysteine and methionine metabolism Metabolic pathways
24	UDP-2-acetamido-2,6-dideoxy-beta-L-talose	C_17_H_27_N_3_O_16_P_2_	2.01	Neg	−2.48	0.03	1.55	O-Antigen nucleotide sugar biosynthesis

## Data Availability

All data generated or analyzed during this study are included in this article. Pathway analysis was performed with the MATLAB scripting language (Mathworks^®^, Natick, MA, USA), using the MetaboAnalyst (http://www.metaboanalyst.ca/) and KEGG (http://www.genome.jp/kegg/) websites, both accessed on 4 March 2022.

## Supplementary Materials

The following supporting information can be downloaded at: https://www.mdpi.com/article/10.3390/ani12070890/s1, Figure S1. Expression profiles of differential metabolites after drought exposure in (a) positive and (b) negative ion mode. Figure S2. Changes in ion intensity potential biomarkers. Figure S3. Survival of crayfish. Table S1. Significantly altered metabolites.
