# Supplementary material for: Changes in the Immunity, Histopathology, and Metabolism of Crayfish (Procambarus clarkii) in Response to Drought
Source: Animals (Basel). 2022 Mar 31;12(7):890. doi: 10.3390/ani12070890 (PMC8996970; doi:10.3390/ani12070890)
Supplement: Supplementary file 1 [file animals-12-00890-s001.zip › Supplementary Materials.pdf]

Supplementary Materials:

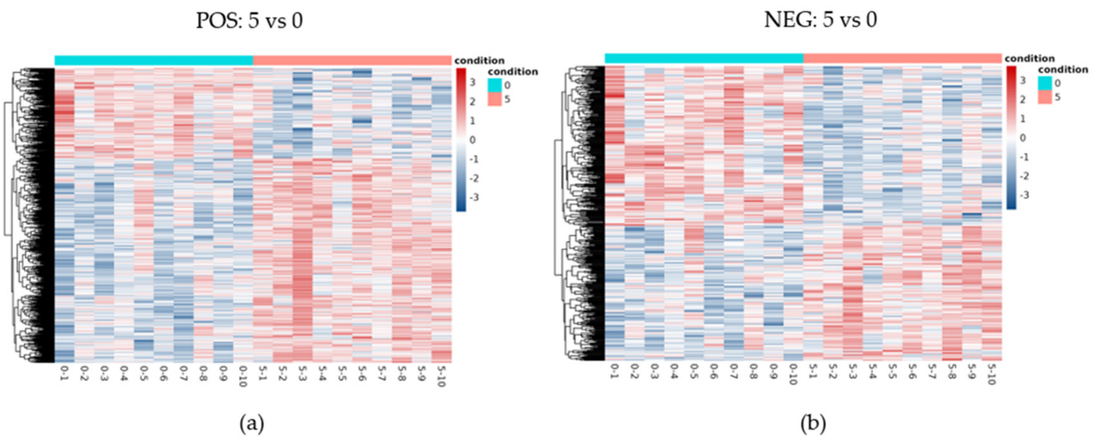

Figure S1. Expression profiles of all differential metabolites after drought exposure in positive(a) and negative(b) ion modes. Colors represent the metabolites expression levels from blue (low) to red (high).

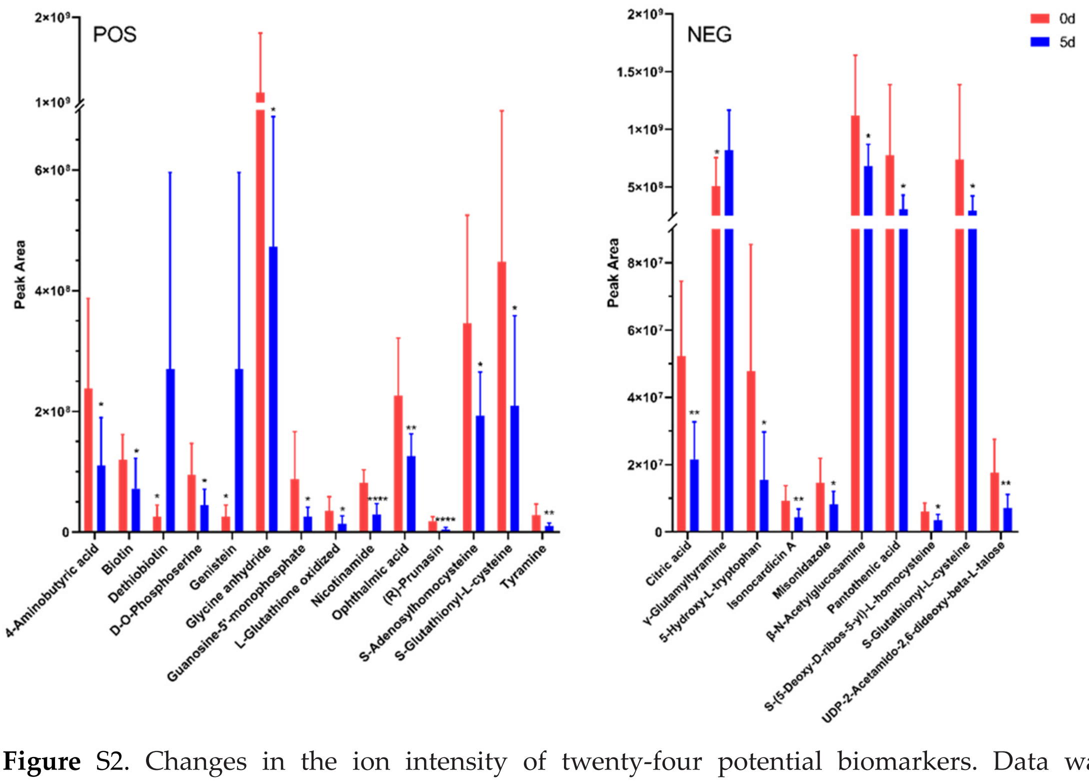

Figure S2. Changes in the ion intensity of twenty-four potential biomarkers. Data was expressed as mean±S.D, n=10. $P < 0.05$, $P < 0.01$ or $P < 0.001$ were considered statistically significant and indicated by *, ** or ***, respectively.

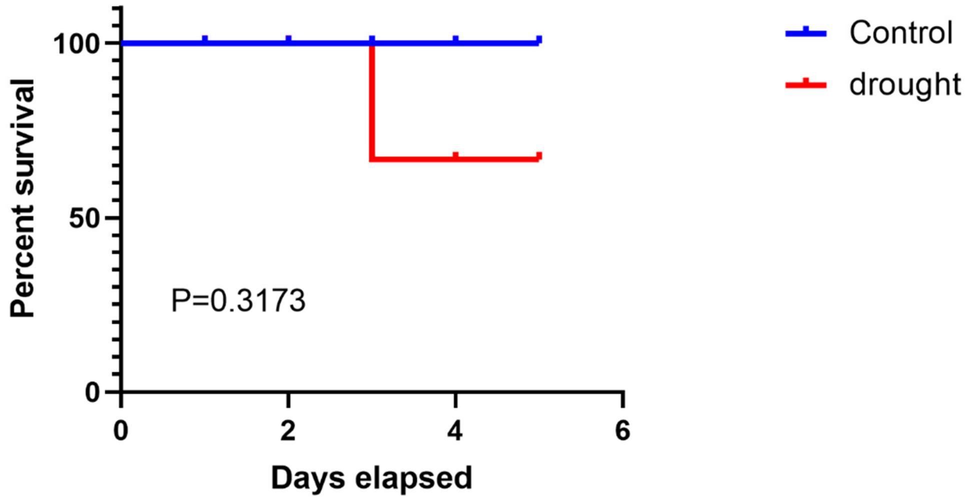

Figure S3. Survival of crayfish.
